# Catalyzing decisions: How a coin flip strengthens affective reactions

**DOI:** 10.1371/journal.pone.0220736

**Published:** 2019-08-14

**Authors:** Mariela E. Jaffé, Leonie Reutner, Rainer Greifeneder

**Affiliations:** 1 Center for Social Psychology, University of Basel, Basel, Switzerland; 2 Department of Pharmaceutical Sciences, University of Basel, Basel, Switzerland; Shandong University of Science and Technology, CHINA

## Abstract

When individuals are undecided between options, they may flip a coin or use other aids that produce random outcomes to support decision-making. Such aids lead to clear suggestions, which, interestingly, individuals do not necessarily follow. Instead when looking at the outcome, individuals sometimes appear to like or dislike the suggestion, and then decide according to this feeling. In this manuscript we argue that such a decision aid can function as a catalyst. As it points to one option over the other, individuals focus on obtaining this option and engage in a more vivid representation of the same. By imagining obtaining the option, feelings related to the option become stronger, which then drive feelings of satisfaction or dissatisfaction with the outcome of the decision aid. We provide support for this phenomenon throughout two studies. Study 1 indicates that using a catalyst leads to stronger feelings. Study 2 replicates this finding using a different catalyst, and rules out alternative explanations. Here, participants report that after having used a catalyst, they experienced a stronger feeling of suddenly knowing what they want compared to the control group that did not use a catalyst. Implications of these results for research and practice are discussed.

## Introduction

Individuals face a multitude of decisions every day. Some are clear-cut, and allow for easy decisions, whereas for others individuals may be torn between options. In some of these cases, individuals might try to find a solution for their dilemma by using a simple random outcome decision-making aid such as a coin. By determining the meaning of heads and tails and by flipping the coin, individuals can easily come to a decision. However, sometimes individuals report a curious phenomenon when looking at the outcome: Despite having been undecided before, suddenly, they experience a feeling of liking or disliking the outcome and use this feeling to decide on their own.

Experiencing sudden feelings regarding the outcome of a coin flip is not an unfamiliar phenomenon. In a short survey that we conducted via Clickworker, all 31 participants were familiar with flipping a coin to make a decision, and 87% indicated that they themselves had applied that strategy in the past. 58% of participants indicated that the coin toss elicited a feeling, such as tension or excitement (32%), but they also reported having felt happy or sad (21%)–depending on the outcome of the coin toss. Apparently, flipping a coin may be more than a decider that shoulders the burden of choice by suggesting a random outcome, but may affect feelings that help individuals to decide on their own.

In this manuscript, we investigate this interesting phenomenon and put it to the test. Throughout two studies we demonstrate that using a decision-making aid with random outcomes, such as a coin flip, strengthens feelings and results in impressions of suddenly knowing what individuals want in the first place.

### A brief overview of coin flips

Coin flips or other decision-making aids with random outcomes have been studied before, but in a more classical situation such as the old standing practice in soccer games to determine which team will play the first half of the game on which side and who will kick the ball first [1]. In this classic situation, the outcome of the coin flip *determines* the decision, and individuals cannot decide on their own.

In this context, Keren and Teigen [1] investigated individuals’ willingness to toss a coin in situations of indeterminacy, where there are no decisive arguments that favor one alternative over another. The authors focus on the finding that for trivial decisions a coin flip is considered an efficient and fair means of making decisions, but when the outcome is more important, a decisive coin toss becomes less acceptable, as this approach seemed to conflict with traditional ideas about argument-based rationality and personal responsibility of the decision maker [1].

In situations that are less dramatic and more irrelevant, individuals may instead choose to use a coin flip to delegate the choice to randomness [2] or to avoid being responsible for implementing inequity [3]. In a more applied setting, Levitt [4] conducted a large field experiment and asked participants about an important decision they needed to make. He then let them flip a coin. After two and six months participants were contacted again and asked about their decision and associated satisfaction. His study revealed that individuals who were told by the coin toss to make a change (compared to maintain the status quo) were much more likely to make the change and were happier six months later.

### Flipping a coin to *help* make a decision

When individuals flip a coin, they do not necessarily need to follow the outcome. Sometimes they report liking or disliking the outcome and this feeling then holds information in itself, as individuals follow the coin’s suggestion, when they like it, but go for another option, when they don’t.

To illustrate this theoretical process, we briefly summarize the story of a friend of one of the authors. This friend once had to make a difficult career decision between two jobs after graduating from his law studies. On the one hand, there was a very well-paid position in a prestigious law firm, which would probably require high commitment and a lot of overtime. On the other hand, there was a position in a smaller company, which was not as well-known and also paid less, but allowed for more flexibility in working hours. Both jobs had distinct advantages and disadvantages, making it difficult to come to a conclusion. The friend drafted lists of pros and cons, thoroughly considered the options, and talked to other people. As these strategies did not allow for a clear decision, he simply tossed a coin in the end. The coin suggested going for the well-paid job with long working hours. He disliked the outcome of the coin flip and *felt* that he actually did not want to take that job. Instead, he decided to take the other job.

But why would a random device, such as a coin flip, lead to feelings of liking or disliking? In brief, we hypothesize that as the coin flip suggests making a certain choice by pointing to one option over the other, the individual focuses on the option the coin is pointing to. More specifically, the individual concretely imagines obtaining this option (taking the prestigious job). This process of mental simulation results in a more vivid representation of the choice. The vivid representation makes previously existing feelings stronger. Like a magnifying glass, the coin flip amplifies positive and negative affect so that previously undetectable differences between positive and negative reactions may become apparent. In our example, these feelings are of negative valence (negative feelings about overtime overpowered the positive ones elicited by the high pay), individuals might dislike the outcome of the coin flip. If, instead, the stronger feelings are of positive valence, individuals may like the outcome of the coin flip. These feelings may then have an impact on subsequent processes, arguably resulting in a clearer preference and decision.

Perhaps the role the random outcome decision-making aid plays in such a situation is best described as a catalyst, since it allows for a decision where individuals were undecided before. We hypothesize that random devices may catalyze decisions by rendering feelings stronger, eventually resulting in more affect-driven judgment and decision processes.

### Why a catalyst may elicit feelings that matter in decision-making

Our novel perspective of decision aids as *catalysts* is different from the classic perspective of using them as *deciders*, as it highlights that decision makers decide on their own, but are informed by feelings strengthened during the coin flip.

We assume when using a decision-making aid such as a coin flip, the suggestion of one option over the other is not binding, but it may feel very real. Therefore, using a decision-making aid may result in a more vivid representation of the suggested option, which may be linked to stronger feelings. This is consistent with previous research, which has shown that emotion is positively linked to mental imagery [5], and that concrete mental simulations evoke stronger emotional reactions than more abstract ones [6]. Moreover, Hsee and Rottenstreich [7] showed that providing more vivid information, such as pictures compared to text, leads to increased salience of feelings.

Strengthened feelings can then serve as pieces of information in their own right. The feeling-as-information account suggests that such feelings, just like pieces of content information, can be used as information to make decisions [8,9]. In accordance with the “How do I feel about it” heuristic [10,11], individuals can inspect the feelings that the representation of a certain target may elicit when being pursued [12]. If the feelings that were strengthened by vividly imagining obtaining the suggested option are positive, individuals can use this information as an indicator for their actual preference. If, however, the feelings that were strengthened by vividly imagining obtaining the suggested option are negative, individuals can use this information to conclude that they might prefer the alternative option. Therefore, using a catalyst may result in an immediate feeling of which option individuals prefer.

In consequence, depending on whether the strengthened feelings are positive or negative towards the suggested option, feelings such as liking or disliking the decision-making aid’s outcome may result. Positive feelings should result in stronger feelings of liking or approving of the coin flip’s suggestion, while negative feelings should result in stronger feelings of disliking or disapproving of the coin flip’s suggestion. Next, these affective reactions towards the decision-making aid can be used to form a clear preference for one option over the other and therefore allow individuals to come to a decision. Suddenly, they may report having a feeling of knowing what they want.

### Summary of studies

To summarize, decision-making aids that produce random outcomes may function not only as deciders (as investigated in other research), but also as catalysts. As decision-making aids point to one option over the other, individuals may vividly imagine and mentally experience obtaining the outcome. By this visual and sensory representation, feelings related to the decision option become stronger and, depending on their valence and the suggestion of the coin flip, result in satisfaction or dissatisfaction with the outcome. Against this background, we suggest:

- Hypothesis 1: Participants using a catalyst (catalyst-participants) compared to participants without a catalyst (control-participants) report stronger affective reactions and feelings of satisfaction (liking) and/or dissatisfaction (disliking)
- Hypothesis 2: These affective reactions are helpful in clarifying one’s preference, and therefore result in a feeling of knowing what one wants.

Two studies that use different catalysts are reported in support of these hypotheses. We received an IRB approval from the IRB of the University of Basel, Faculty of Psychology, with the approval number 001–14 and obtained written consent in both online studies. All data and materials are available under: https://drive.switch.ch/index.php/s/1HzUVPqmdhT1HSq.

## Study 1

Study 1 investigates whether a catalyst may strengthen feelings, which individuals consciously experience and report. During the study, participants made five decisions and tossed a coin before each. Half of the participants were asked to ignore the outcome (control condition which serves as baseline), and half were asked to think of the coin as a decision aid (catalyst condition). We hypothesized that feelings would be strengthened for catalyst-participants, as receiving a recommendation and not simply looking at a coin flip is the crucial ingredient when a decision making aid serves as a catalyst. Moreover, we hypothesized that catalyst- compared to control-participants would report more disapproval and approval as well as more dissatisfaction and satisfaction with the coin’s outcome (depending on whether the coin toss pointed to an option that felt *bad* versus *good*). Complementing the picture, we further hypothesized that control- compared to catalyst-participants would report more indifference regarding the coin’s outcome. Importantly, Study 1 aimed at testing the general hypothesis of whether using a catalyst strengthened feelings. We did not intend to disentangle the valence of the feelings (negative or positive) as we believe that the coin flip could lead to stronger feelings of satisfaction *or* dissatisfaction and both affective reactions would be in support of our hypothesis.

### Method

#### Participants

The study was conducted online, advertised as *Visit to a restaurant*: *Choose your menu* on the platform Clickworker, and took about eight to nine minutes to complete. Assuming a medium effect, and setting alpha to .05 and power to .80, we aimed to recruit 40 participants per cell [13]. Seventy-eight individuals participated (38 males, 40 females; *M*_*age*_ = 35.76 years, *SD*_*age*_ = 11.35). Participants received 0.75€ (approximately US$0.75) as compensation.

#### Design

Throughout the study participants chose five courses of a restaurant meal. For each course they were asked to decide between two options. Before making the decision a coin was tossed. As a between factor we either asked participants to use the coin toss as a decision-making aid that randomly suggests an option (catalyst-condition) or we labeled the coin toss as irrelevant and asked participants to ignore it (control-condition).

#### Materials and procedure

Participants gave written informed consent and could only participate in the study if they indicated being at least 18 years old and had read and understood the consent form. Also, they were first asked to indicate any specific dietary preferences (e.g., being a vegetarian or eating gluten-free). We used this information to tailor the menu options such that all participants could eat what they were offered as choices. Subsequently, participants were introduced to the coin toss, which was integrated into the study as an animated GIF-image of a spinning coin that stopped randomly at either heads or tails. Participants could give the coin toss as many tries as they wanted. This was done to increase their faith in a random process. In the catalyst-condition, participants learned that heads meant the coin suggested the option on the left and tails meant the coin suggested the option on the right. In the control-condition, participants were asked not to let the coin distract them from making their decisions.

All participants then proceeded to the decision about their first course. Two options were offered (e.g., tuna carpaccio with fresh horseradish versus quinoa-pesto salad with nut-bread), one on the left and one on the right side of the screen. The coin would then be tossed and either pointed to one option (therefore making a suggestion; catalyst-condition) or appeared in the middle of the screen (therefore making no suggestion; control-condition). Subsequently, participants were asked to decide by clicking on one of the options. This procedure was repeated for all five courses of the menu.

After choosing the fifth course, participants were asked how much attention they had paid to the outcome of the coin toss (heads or tails) (1 = *not at all*; 7 = *very much*) and how much they had been interested in the outcome of the coin toss (heads or tails) (1 = *not at all*; 7 = *very much*); these items served as manipulation checks. Participants were then asked to which extent they experienced a reaction of (a) indifference, (b) annoyance, (c) disapproval and/or approval to the outcome of the coin toss, and (d) dissatisfaction and/or satisfaction as a reaction to the coin tosses (1 = *not at all*; 7 = *very much*). At the end, participants were asked if, immediately after the coin tosses, they had experienced a feeling regarding which option they would (not) prefer (1 = *no feeling at all*; 7 = *very strong feeling*).

Finally, participants were asked to provide demographic information including gender and age, and to indicate how diligently they had completed the questionnaire. Participants were thanked and received their compensation.

### Results

To compare answers of participants in the catalyst-condition and control-condition, we calculated t-tests for independent samples. When a significant Levene test indicated that variances were not equal, we report the corrected results.

#### Manipulation check

Catalyst-participants indicated having paid more attention to the outcomes of the coin toss compared to control-participants, *M*_*catalyst*_ = 3.26, *SD*_*catalyst*_ = 1.96, and *M*_*control*_ = 2.05, *SD*_*control*_ = 1.57, respectively, *t*(72.63) = 3.00, *p* = .004, *r* = 0.33. Catalyst-participants were also more interested in the coin toss’ outcome compared to control-participants, *M*_*catalyst*_ = 2.51, *SD*_*catalyst*_ = 1.75, and *M*_*control*_ = 1.79, *SD*_*control*_ = 1.36, respectively, *t*(71.73) = 2.03, *p* = .046, *r* = 0.23.

#### Feelings

In support of our hypotheses, catalyst- compared to control-participants felt more strongly about which option they would prefer immediately after the coin tosses, *M*_*catalyst*_ = 3.85, *SD*_*catalyst*_ = 2.17, and *M*_*control*_ = 2.10, *SD*_*control*_ = 1.70, *t*(71.84) = 3.95, *p* < .001, *r* = 0.42. Moreover, catalyst- compared to control-participants reported stronger feelings of disapproval and/or approval (*M*_*catalyst*_ = 4.08, *SD*_*catalyst*_ = 1.68, and *M*_*control*_ = 1.92, *SD*_*control*_ = 1.38, *t*(76) = 6.19, *p* < .001, *r* = 0.58) as well as dissatisfaction and/or satisfaction (*M*_*catalyst*_ = 3.49, *SD*_*catalyst*_ = 1.60, and *M*_*control*_ = 2.51, *SD*_*control*_ = 1.55, *t*(76) = 2.73, *p* = .008, *r* = 0.30).

In contrast, compared to catalyst-participants, control-participants indicated more indifference regarding the coin toss (*M*_*catalyst*_ = 4.51, *SD*_*catalyst*_ = 1.81, and *M*_*control*_ = 5.77, *SD*_*control*_ = 1.74), *t*(76) = -3.13, *p* = .002, *r* = 0.34. No statistically significant differences occurred regarding annoyance (*M*_*catalyst*_ = 4.13, *SD*_*catalyst*_ = 1.64, and *M*_*control*_ = 4.74, *SD*_*control*_ = 1.96), *t*(76) = -1.51, *p* = 136, *r* = 0.17.

Overall, we find that the strength of feelings was also correlated with the strength of other feelings or impressions. We find that feelings of disapproval and/or approval were positively correlated with feelings of dissatisfaction and/or satisfaction, *r*(78) = .73, *p* < .001 and interest in the coin (see manipulation check) was negatively correlated with feelings of indifference, *r*(78) = -.34, *p* = .002.

#### Additional analyses

To test whether catalyst-participants simply followed the coin’s suggestion when making their choices, we analyzed the occurrence of dependencies between the outcome of the coin toss and the choices made by calculating a χ^2^-Tests for each of the five menu courses. None of these tests indicates dependencies (all χ^2^s < 1), except for course four, where a slight tendency for choosing the option the coin was not pointing to was observed, χ^2^(1) = 3.30, *p* = .069. Overall, these results suggest that participants did not use the coin in the traditional sense of a decider, but as a catalyst.

#### Exploratory analyses regarding decision times

Research suggests that individuals can be very fast when making decisions [14]. Nevertheless, it appeared interesting to exploratorily investigate whether strengthened feelings might also translate to differences in decision times. These analyses however need to be treated with caution, as our experimenting program (Unipark by questback) records reaction time estimates on the server and not client, so that reaction times can be heavily influenced by participants’ internet connection as well as other server activity. Furthermore, in a minority of cases the software mistakenly results in negative reaction times. If this was the case, we excluded these data points from our analyses. The reported reaction times may therefore be best understood as approximations of the actual response times. Moreover, as the distributions of the response time measures are skewed, we calculated the median response time for every course and compared these statistics across conditions with an Independent-Samples Median Test.

Analyses of these reaction times suggest a significant difference in the medians of the catalyst versus control condition for course number one, but no significant difference in the medians for courses two to five, *Mdn1*_*catalyst*_ = 8.00, *Mdn1*_*control*_ = 6.50, *p* = .030; *Mdn2*_*catalyst*_ = 5.00, *Mdn2*_*control*_ = 5.00, *p* = .937; *Mdn3*_*catalyst*_ = 4.00, *Mdn3*_*control*_ = 4.00, *p* = .306; *Mdn4*_*catalyst*_ = 4.00, *Mdn4*_*control*_ = 4.00, *p* = .887; *Mdn5*_*catalyst*_ = 4.00, *Mdn5*_*control*_ = 4.00, *p* = .743.

We also exploratorily investigated whether decision times in the catalyst condition might differ for trials in which the suggestion of the coin flip and the participants’ decision aligned (are congruent) versus did not align (are incongruent). Here, we find no significant difference in the medians for decision making time depending on whether choices were congruent or incongruent with the coin flips’ suggestion, *Mdn1*_*congruent*_ = 9.00, *Mdn1*_*incongruent*_ = 8.00, *p* = .344; *Mdn2*_*congruent*_ = 5.00, *Mdn2*_*incongruent*_ = 4.50, *p* = .910; *Mdn3*_*congruent*_ = 4.00, *Mdn3*_*incongruent*_ = 4.00, *p* = .733; *Mdn4*_*congruent*_ = 4.00, *Mdn4*_*incongruent*_ = 4.00, *p* = .869; *Mdn5*_*congruent*_ = 4.00, *Mdn5*_*incongruent*_ = 4.00, *p* = .746.

### Discussion

Study 1 provides first experimental evidence that flipping a coin may strengthen feelings. Specifically, catalyst- compared to control-participants reported stronger immediate feelings of which option they would (not) prefer. Furthermore, catalyst-participants reported stronger feelings of disapproval versus approval and dissatisfaction versus satisfaction concerning the outcome of the coin. Complementarily, control-participants were more indifferent to the outcomes of the coin tosses.

## Study 2

Study 1 revealed that a decision aid used as a catalyst (compared to just being present yet ignored) strengthens feelings. This finding supports our hypothesis and sheds light on the interesting phenomenon of why individuals might find it helpful to flip a coin when making a decision even if they did not simply follow its suggestion (as the data from Study 1 suggests). After looking at the results from Study 1, however, some questions remain open. As we were generally interested in strengthened feelings, we did not differentiate for the feeling’s valence, but combined satisfaction and dissatisfaction into one item. It would nevertheless be interesting to see whether feelings of satisfaction might have been more likely than feelings of dissatisfaction or vice versa. Study 2 was set up to allow us to investigate the valence of the feelings elicited by using a catalyst.

Furthermore, at least two alternative explanations may be advanced and need addressing. First, it could be argued that control-participants felt discouraged from reporting feelings, because reporting feelings might indicate that they did not properly follow instructions, that is, ignore the coin tosses. To formally address this account, Study 2 was designed in such a way that all participants had to pay attention to the catalysts. This change also addresses a second alternative account, which holds that control- compared to catalyst-participants were distracted by the ignoring-task and therefore paid less attention to their feelings.

Compared to Study 1, two further changes were introduced to better understand the phenomenon. First, we additionally assessed feelings directly after each decision and did not ask participants for a summary judgment at the end of the study, which they might find difficult to generate and which therefore might be less informative. Second, we introduced a different catalyst to demonstrate generalizability.

### Method

#### Participants

The study was conducted as an online study and advertised as *Visit to a restaurant*: *Choose your menu* on the platform Clickworker and took about eight minutes to complete. Taking into account that Study 1 revealed small to medium effects, and setting alpha to .05 and power to .80, we aimed to recruit 90 participants [13]. Eighty-seven individuals participated (47 males, 38 females, 2 no answer; *M*_*age*_ = 35.97 years, *SD*_*age*_ = 12.39). Participants received 0.90€ (approximately US$0.90) as compensation.

#### Design

As in Study 1, participants hypothetically chose five meal courses in a restaurant. For each course they were asked to decide between two options and all participants were always provided with the decision aid. The variable of interest was whether each trial included a suggestion (catalyst-trial) versus not (control trial), which was manipulated as a within factor *across* the five trials.

Importantly, we varied the number of times from zero to five that participants received a suggestion from the aid, producing zero to five catalyst- versus control trials for every participant. Number of suggestions (zero to five) was treated as a between subjects factor, and participants were randomly assigned to one of the six factor levels.

#### Materials and procedure

The study used the same menu options as Study 1. Participants received a link to the online study, gave written informed consent (see Study 1 for further details), and learned from the instructions that they would be choosing a five-course meal. Participants were then introduced to a die that served as a decision-making aid, which would be rolled before each decision. Crucially, if the die outcome was a one or two, it suggested choosing the left or the right option, respectively, thus serving as a catalyst (catalyst-trial). In contrast, if the die outcome was a three, four, five, or six, the die did not make a suggestion (control-trial). Participants could make as many test rolls of the die as they wanted.

Subsequently, participants proceeded to the decision about their first course. Two options were offered, one on the left and the other on the right side of the screen. The die would then be rolled above these options (see S1 Appendix for screenshots) and either indicated a suggestion by appearing above one option (catalyst-trial) or in the middle of the screen below the two options (control-trial). Participants were then asked to make a decision by clicking on one of the options. After making their choices, participants were asked if the die had suggested one of the menu options (*yes* vs. *no*, as manipulation check). We also asked participants to indicate which of the following would best describe their reaction right after the die roll: *satisfaction*, *dissatisfaction*, *indifference*, or *annoyance* (forced-choice item). Finally, we asked if rolling the die elicited an immediate feeling of which option they would prefer or not prefer (1 = *no feeling at all*; 7 = *very strong feeling*). This procedure was repeated for all five courses, meaning that in Study 2 participants rated their feelings after each (non)recommendation of the die.

After choosing the final course, participants were prompted for several summary judgments. In particular, we asked how much they were interested in the outcomes of rolling the die (1 = *not at all*; 7 = *very much*) and if the die elicited an immediate feeling of which option they would prefer or not prefer, separately for catalyst-trials and control-trials (1 = *no feeling at all*; 7 = *very strong feeling*). We also asked participants if, while rolling the die, they had wished that it would point to one option over the other (*never* to *five times*, coded 1–6 in the analysis). At the end of the survey, participants were asked to provide demographic information including gender and age. Participants were thanked and received their payment.

### Results

To check whether participants understood the set-up, we analyzed whether participants indicated that the die made a suggestion when it actually pointed to one option over the other (manipulation check). Out of 87 participants, 77 answered these questions correctly for all five trials, eight individuals made one mistake, one participant made two mistakes, and one made four mistakes. We checked whether excluding the participants changed the pattern of the results, which was not the case. We thus report the results for the entire sample.

#### Strengthening feelings

Looking at the question of whether catalyst-trials elicited an immediate feeling of which option participants would (not) prefer, we calculated a mixed model [15] with type of trial as an independent and strength of an immediate feeling of preference as a dependent variable, with trial and participants as random factors. Participants reported having stronger immediate feelings in catalyst- compared to control-trials, *M*_*catalyst*_ = 2.65, *SD*_*catalyst*_ = 1.82, and *M*_*control*_ = 1.95, *SD*_*control*_ = 1.54, *t*(34.91) = -3.20, *p* = .003.

#### Frequency of feelings

To analyze whether emotional reactions towards the die outcomes differ depending on catalyst- versus control-trials, we collapsed the results of the five rounds and calculated χ^2^ Tests with type of trial as an independent and the resulting feeling as dependent variable. There was a significant association between condition and the reported feeling χ^2^ (3) = 30.17, *p* < .001. For catalyst trials, 23.3% of participants indicated a reaction best described as satisfaction, 9.6% as dissatisfaction, 57.1% as indifference, and 10% as annoyance. In contrast, in control-trials, only 6.5% of participants indicated reacting in a way best described as satisfaction, 6.9% as dissatisfaction, but 66.7% indicated indifference and 19.9% annoyance. In catalyst- compared to control-trials, participants were 3.6 times more likely to indicate satisfaction and 1.4 times more likely to report dissatisfaction as the predominant reaction. In control- compared to catalyst-trials, participants were 1.15 times more likely to indicate indifference and 1.96 times more likely to indicate annoyance as best describing the reaction elicited by the outcome of the die roll. Analyzing standardized residuals, we can further conclude that feelings of satisfaction were reported significantly more often in catalyst-trials, and significantly less often in control-trials, than would be expected by chance, *z* = 3.2 and *z* = -3.2, respectively; all other standardized residuals, *z*s < |1.91|.

#### Frequency of recommendations

To check whether the number of suggestions (zero to five) is associated with the frequency of the specified feelings, we calculated bivariate correlations. Interestingly, the number of recommendations correlated significantly positively with frequency of satisfaction, *r*(87) = .26, *p* = .016, and significantly negatively with frequency of annoyance, *r*(87) = -.26, *p* = .016, meaning that the more recommendations the die made, the more feelings of satisfaction and less feelings of annoyance were reported. The remaining correlations were not significant, *r*s < |.13|.

#### Summary evaluations after making all decisions

To compare the strength of immediate feelings of (not) preferring an option for catalyst- compared to control-trials (two separate questions), we calculated a paired samples t-test. For catalyst-trials, participants indicated stronger immediate feelings than for control-trials, *M*_*catalyst*_ = 2.32, *SD*_*catalyst*_ = 1.74, and *M*_*control*_ = 1.74, *SD*_*control*_ = 1.40, respectively, *t*(56) = 2.64, *p* = .011, *r* = 0.33. Furthermore, to check for alternative explanations we analyzed whether participants differed in their interest in the outcome of the die roll, depending on having received more or fewer suggestions. We calculated a linear regression to predict interest in the outcome of the die roll based on the number of suggestions, which was not significant, *F*(1, 86) = 1.38, *p* = .244.

#### Exploratory analyses on strengthening feelings

As in Study 1, we investigated how often participants’ decisions were in line with the coin flip. For each round we coded whether the decision was congruent with the die’s suggestion (coded as 1) or incongruent with the die’s suggestion (coded as 0). We then tested whether the average congruency score differed from chance level of 0.5. Across the five courses none of the tests yielded significant results: *t*_*Course1*_(42) = 1.39, *p* = .173; *t*_*Course2*_(41) = -1.91, *p* = .063; *t*_*Course3*_(41) = 1.24, *p* = .221; *t*_*Course4*_(48) = 1.00, *p* = .322; and *t*_*Course5*_(42) = -0.15, *p* = .881. These results suggest that participants did not automatically just go with the cue but made an independent decision.

Furthermore, we exploratorily investigated whether feelings are stronger in catalyst trials when participants’ choices were congruent with the die’s recommendation or not. To this end we calculated an independent t-test for every course with congruency as an independent variable (0 = incongruent, 1 = congruent) and strength of immediate feeling as a dependent variable. For course number one, strength of feelings was *M*_*congruent*_ = 3.23, *SD* = 2.10; *M*_*incongruent*_ = 2.47, *SD* = 1.63; *t*(39.74) = -1.33, *p* = .190. For course number two, strength of feelings was *M*_*congruent*_ = 4.07, *SD* = 2.15; *M*_*incongruent*_ = 2.33, *SD* = 1.69; *t*(40) = -2.89, *p* = .006. For course number three, strength of feelings was *M*_*congruent*_ = 2.44, *SD* = 1.98; *M*_*incongruent*_ = 2.76, *SD* = 1.68; *t*(40) = 0.55, *p* = .583. For course number four, strength of feelings was *M*_*congruent*_ = 2.68, *SD* = 1.70; *M*_*incongruent*_ = 1.95, *SD* = 1.12; *t*(46.31) = -1.80, *p* = .078. For course number five, strength of feelings was *M*_*congruent*_ = 2.62, *SD* = 2.13; *M*_*incongruent*_ = 2.32, *SD* = 1.34; *t*(41) = -0.55, p = .585. All in all, these results suggest a significant difference in strength of feelings only for course number 2. It can thus not be concluded that stronger feelings occurred when individuals’ choices were congruent versus incongruent with the die’s suggestion.

#### Exploratory analyses regarding decision times

We exploratorily investigated whether strengthened feelings might also translate to differences in decision times. These analyses however need to be treated with caution; see comments in Study 1. No significant difference in the medians between catalyst and control trials for each of the different courses was observed, *Mdn1*_*catalyst*_ = 8.00, *Mdn1*_*control*_ = 9.00, *p* = .738; *Mdn2*_*catalyst*_ = 4.00, *Mdn2*_*control*_ = 4.00, *p* = .970; *Mdn3*_*catalyst*_ = 4.00, *Mdn3*_*control*_ = 4.00, *p* = .657; *Mdn4*_*catalyst*_ = 4.00, *Mdn4*_*control*_ = 3.00, *p* = .357; *Mdn5*_*catalyst*_ = 4.00, *Mdn5*_*control*_ = 4.00, *p* = .705; all tests are Independent-Samples Median Tests.

We also exploratorily investigated whether decision times in the catalyst condition might differ for trials in which the suggestion of the coin flip and the participants’ decisions aligned (are congruent) versus did not align (are incongruent). Here, we find no significant difference in the medians for decision making time depending on whether choices were congruent or incongruent with the coin flips’ suggestion, *Mdn1*_*congruent*_ = 9.00, *Mdn1*_*incongruent*_ = 7.00, *p* = .617; *Mdn2*_*congruent*_ = 3.00, *Mdn2*_*incongruent*_ = 5.00, *p* = .092; *Mdn3*_*congruent*_ = 4.00, *Mdn3*_*incongruent*_ = 4.00, *p* = .871; *Mdn4*_*congruent*_ = 4.00, *Mdn4*_*incongruent*_ = 4.00, *p* = .967; *Mdn5*_*congruent*_ = 4.00, *Mdn5*_*incongruent*_ = 4.00, *p* = .920.

### Discussion

Study 2 was conducted to replicate the findings from Study 1 with a different catalyst, a die, to gauge the results’ generalizability. Furthermore, Study 2 was designed to rule out alternative explanations. This was achieved by providing all participants with the decision aid in all trials, yet varying whether the aid provided a suggestion and thus served as a catalyst (catalyst-trials) or not (control-trials).

Results of Study 2 corroborate the hypotheses as participants reported stronger feelings in catalyst- compared to control-trials. Moreover, feelings of satisfaction were reported significantly more often in catalyst-trials, and significantly less often in control-trials, than would be expected by chance. Furthermore, the more suggestions participants received, the more feelings of satisfaction were reported, although our exploratory analyses show that participants did not simply followed the die’s suggestion. This indicates that receiving a recommendation could strengthen feelings but would not necessarily determine the decision. The more suggestions participants received, the fewer feelings of annoyance were reported. This could indicate that using a die that did not recommend an option and therefore was useless was annoying for participants.

Interestingly, feelings of dissatisfaction did not significantly vary between catalyst- and control-trials. At first glance, this appears to be in conflict with our hypothesis that when the die points to the option that feels wrong, dissatisfaction arises. However, dissatisfaction may also arise when the aid does not make a suggestion at all. As a result, dissatisfaction may be present in both catalyst- and control-trials, but for different reasons.

## General discussion

Previous research has investigated using decision-making aids with random outcomes, such as a coin toss or die, which function as a *decider* by determining the choice for the individual. Here we offer a novel perspective suggesting that decision-making aids might function as *catalysts* that strengthen feelings. Out of all possible options, the catalyst suggests a choice, which is not binding, but nevertheless can feel very real. As a result, individuals may imagine the decision options more vividly, which in turn elicits stronger positive or negative affective reactions. These positive and negative reactions can then be helpful in clarifying one’s preferences and knowing which option one wants to choose.

Two studies were designed to test this novel notion of catalyzing decisions. In both studies, participants were asked to make hypothetical choices between two dishes each for a five-course menu. Here, we ensured that all participants could eat what they were presented with, that the menus sounded fancy and extraordinary, and that participants were not used to the dishes they chose from. As such, we believe that there is good reason to assume that the decisions were not easy. In this context, Study 1 provides first experimental support that flipping a coin to make such menu choices may strengthen feelings. Specifically, catalyst- compared to control-participants reported stronger immediate feelings of which option they would (not) prefer. Study 2 replicates Study 1 with a different catalyst, and furthermore rules out alternative explanations. Study 2 further demonstrates that individuals relying on catalysts more often report having an immediate feeling of knowing what they prefer.

Together, these studies show that relying on decision aids may elicit feelings, which may better inform individuals about their own preferences, resulting in the feeling of immediately knowing what one wants.

### Limitations and options for future research

Studies 1 and 2 provide support for our hypothesis, but leave room for improvement for a set of future research studies. Throughout our work, we aimed to find a particularly strong manipulation of a catalyst by either showing a spinning coin or a rolling die. To further illustrate the suggestion that the device made, the device was displayed above the option it was suggesting. This operationalization however leads to a confound between catalyst trials and alignment of the device above one of the decision options (while in control trials the device is not positioned above one of the decision options). Although we doubt that the simple alignment elicits a stronger immediate feeling of knowing what one wants, it could direct participants’ attention to the decision options, which could alternatively explain why they might have had stronger emotional reactions. Future research might use different visualizations of catalysts that resolve this confound. Having said this, we speculate that the menu options’ exclusiveness captured participants’ attention regardless of whether the catalyst pointed to the options or not.

In our research we investigated the impact of using decision making aids on affective reactions and argue that the changes in affective reactions impact decision making processes. However, one could also wonder whether flipping a coin and looking at its suggestion may trigger more cognitive reactions, too. As we hypothesize that a coin flip leads individuals to imagine the options more vividly, one could assume that they might also think about them in more concrete (instead of abstract) terms. Construal Level Theory [16,17] provides a framework that highlights differences in weighing information when construing options more concretely or abstractly. As an example, individuals have been shown to weigh feasibility considerations more when construing concretely, while desirability considerations are weighed more strongly when construing abstractly [18]. This reasoning could have implications for the usage of decision making aids as well, as a catalyst may trigger differential weighing of attributes (e.g., prestige versus convenience of a job). The present studies do not allow for testing differences in weighing. Future research may therefore fruitfully explore this more cognitive pathway.

Next to investigating the more cognitive side of catalyzing processes, one could also speculate to what extent decision difficulty moderates whether flipping a coin is perceived as helpful or not. Moreover, it may prove interesting to systematically gauge the impact of prior preferences.

Future research could also investigate how personality traits or states might affect the consequences of using a catalyst for decision making. As an example, studies could include a trait measure such as tendency to feel regret [19], analyzing whether individuals who might find it more difficult to make decisions due to anticipated regret might benefit from using a catalyst. Moreover, studies could also focus on the potential regret participants could experience in a certain decision scenario, investigating whether potential regret (just as with decision difficulty) might moderate the catalyzing effects of the random decision making aids.

### Why does affect matter in judgment and decision-making processes?

The aim of the present research was to investigate whether using a catalyst can result in feelings of satisfaction and dissatisfaction. Going beyond the scope of the studies presented here, it is interesting to speculate about downstream consequences of these elicited feelings. Study 2 hints at this question as the results show that catalyst trials were not only associated with stronger affective reactions, but also with an immediate feeling of preferring one option over the other. Apparently, the elicited feelings may allow individuals to form a clear preference and therefore also impact subsequent decision-making processes.

The role of affect in judgment and decision-making has long been investigated and is the topic of thriving research. A plethora of findings suggests that feelings are used in decision-making, and may even constitute a critical ingredient [20–25]. Extant research suggests that reliance on feelings (next to thoughts) in judgments and decisions is not a phenomenon confined to very limited circumstances, but rather the default operation of everyday life [26,27]. Furthermore, reliance on feelings can actually increase under uncertainty [28], which is often the reason for why individuals rely on decision-making aids in the first place.

As described before, the feeling-as-information account suggests that feelings, just like pieces of content information, can be used as information to make decisions [8,9]. This account might be best illustrated by the “How do I feel about it” heuristic [10,11]. This heuristic involves holding a representation of a target in mind and inspecting the feelings that this representation may elicit [12], when being pursued. These feelings, in turn, are used to decide.

### Should individuals use a catalyst to aid decision making?

We have shown that in the setting of asking individuals to make inconsequential menu choices, using a catalyst strengthens feelings and results in an immediate feeling of knowing what to choose. We carefully speculate that the same strategy may successfully be applied to more consequential decision context, too, where helping individuals to make difficult decisions may prove beneficial both on the individual and on the societal level. For instance, choosing a financial savings plan for retirement is an important decision. Individuals, however, often postpone such a decision. However, not making the decision results in a reduced amount of savings for the individuals, as interest and compound interest are forfeited, and there are higher risks for society, as the public may need to cover gaps in individual saving plans. Moreover, not making the decision may produce a very aversive and unpleasant state. If using a catalyst enables individuals to assess their feelings, get information about their preference, and eventually get started on difficult decisions, they and society may benefit on the levels of finance and well-being.

Although we think that there is good reason for these speculations to prove true, it is important to note that the specific circumstances under which the use of a catalyst can be beneficial await further empirical investigation. We assume that using a catalyst strengthens feelings, and others have suggested that relying on feelings can influence and even improve decision-making [23]. However, it is important to note that feelings can serve as a basis of accurate as well as mistaken inferences, depending on the relationship between feelings and the target [26]. Feelings may prove beneficial, for instance, when sufficient background knowledge is possessed, in that individuals who trust their feelings can predict outcomes of future events better than individuals with lower trust in feelings [29]. In sum, if decision quality benefits from including feelings, using a catalyst could be advantageous.

Interestingly, having a strong feeling about one’s own preferences could have benefits that go beyond decision accuracy. An immediate feeling could also increase certainty and perceived ease of deciding. Reversely, post-decisional regret and dissonance may be decreased. Speculating further, decision certainty or ease could even constitute one reason as to why individuals use a decision-making aid with a random outcome in the first place: although they do not know if their decision benefits from using a catalyst, considering their feelings may ease the decision process and increase certainty in regard to the outcome. Further research should investigate the nuances in affective and cognitive reactions towards the usage of catalysts in different decision-making scenarios.

## Conclusion

When individuals are undecided between options, they may flip a coin or use other aids that produce random outcomes to support decision-making. Such aids lead to clear suggestions, but when looking at the outcome, individuals sometimes appear to like or dislike the suggestion, and then decide according to this feeling (and not according to the aid’s suggestion). In this manuscript we present the novel perspective that such a decision aid can function as a catalyst in the decision-making process. Individuals focus on obtaining the suggested option and presumably engage in a more vivid representation of the same, which in turn strengthens affective reactions towards the suggested option and triggers liking or disliking of the decision aid’s suggestion.

## Supporting information

S1 Appendix Screenshots of setup in Study 2. Exemplary screenshots of setup in Study 2. The images and animation of the die roll have been created with the code from https://codepen.io/tameraydin/pen/CADvB?editors=1111, Copyright 2019, Tamer Aydn.
(PDF)
