# Supplementary material for: Catalyzing decisions: How a coin flip strengthens affective reactions
Source: PLoS One. 2019 Aug 14;14(8):e0220736. doi: 10.1371/journal.pone.0220736 (PMC6693849; doi:10.1371/journal.pone.0220736)
Supplement: S1 Appendix — Exemplary screenshots of setup in Study 2. The images and animation of the die roll have been created with the code from https://codepen.io/tameraydin/pen/CADvB?editors=1111, Copyright 2019, Tamer Aydn. (PDF) [file pone.0220736.s001.pdf]

Supporting Information

S1 Appendix. Screenshots of setup in Study 2.

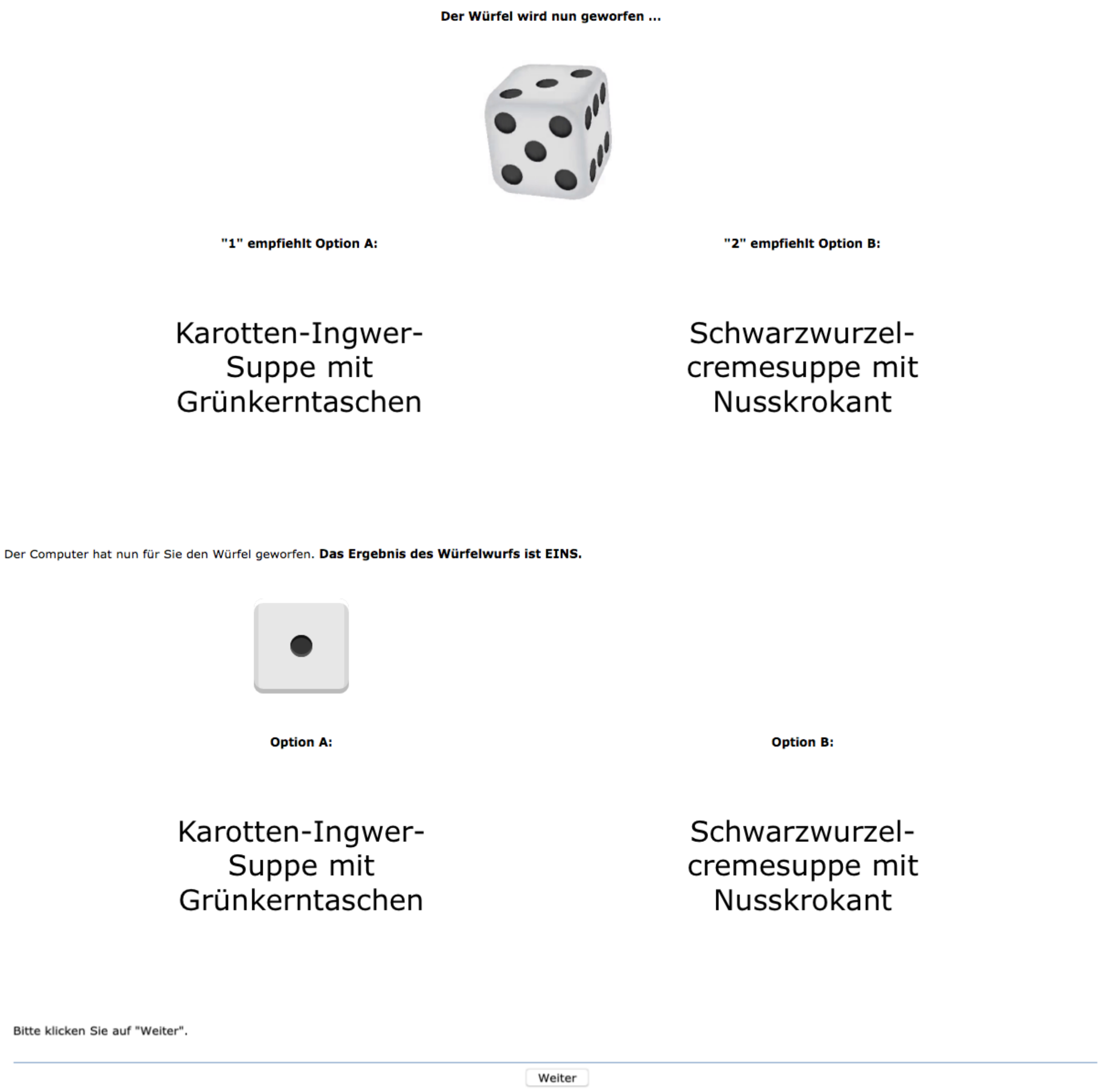

Exemplary screenshots of setup in Study 2. The images and animation of the die roll have been created with the code from https://codepen.io/tameraydin/pen/CADvB?editors=1111, Copyright 2019, Tamer Aydn.
